# Benoit Arsenault on lifestyle, lipids and social media

**DOI:** 10.4155/fso.15.8

**Published:** 2015-11-01

**Authors:** Benoit Arsenault

**Affiliations:** 1 Centre de recherche de l'Institut universitaire de cardiologie et de pneumolgie de Québec, Canada

## Abstract

**Benoit Arsenault speaks to Francesca Lake (Managing Editor, *Future Science Open Access*).** Dr Benoit Arsenault obtained his doctoral degree in physiology–endocrinology from Université Laval in Québec City, Canada in 2009. After two postdoctoral fellowships performed at the Academic Medical Center in Amsterdam (The Netherlands) and at the Montreal Heart Institute (Canada), he became Assistant Professor at the Department of Medicine at Université Laval in 2013. Dr. Arsenault is also a research scientist in the cardiology axis at the Quebec Heart and Lung Institute in Canada.

## Abstract (lay abstract)

The research of Dr. Arsenault's team is focused on high-density lipoprotein (HDL) metabolism, lipoprotein(a), PCSK9, lipid-lowering therapy, atherosclerosis, aortic stenosis and other aspects of the lifestyle-related and inherited risk factors for cardiovascular disease and Type 2 diabetes. Dr. Arsenault is a Senior Editor of *Future Science Open Access*.

## Q What sparked your interest in endocrinology?

I would say that it started when I was an undergraduate in biochemistry in the early 2000s. I absolutely loved energy metabolism classes, both from a cellular and human perspective. At that time, I knew that if I were to graduate studies, I would do a PhD on this topic; I was very fortunate to work for 5 years with Professor Jean-Pierre Després (Université Laval) during my PhD in physiology–endocrinology, who is a world expert in energy metabolism and obesity.

## Q What led to your current specialties in lipidology?

My PhD thesis and a very important part of my current research is focused on the health consequences of obesity and, particularly, abdominal obesity. We have known for some time now that independently of their total body weight or their BMI, many individuals with abdominal obesity and poor fitness levels have a severely impaired lipoprotein and lipid metabolism, which is characterized by high levels of triglycerides and low levels of HDL-cholesterol, often referred to as the ‘good’ cholesterol. A lot of my research nowadays focuses on HDL-cholesterol and the functional aspects of HDL particles.

It has been estimated that abnormal lipid levels account for as much as 50% of heart attacks in most countries around the globe. So, I would say that this, combined with the fact that we have in the province of Québec more than 40 years of experience in lipid research and very productive and talented researchers is why I do research in lipidology.

## Q Can you tell us a little about your current research?

The objective of my research career is nothing short of being part of the generation of scientists and health professionals that will eradicate chronic diseases such as cardiovascular diseases (CVDs) and Type 2 diabetes. As a starting point, I believe that in order to reach this objective, we have to be able to identify and target each and every risk factor for these diseases. An increasing amount of literature suggests that up to four out of five cases of myocardial infarction could be prevented by having a healthy lifestyle. Although it is not too easy to define precisely what exactly is considered a healthy lifestyle, I think we can safely say, based on the scientific evidence that is available, that exercising or being physically active at least 150 min per week, drinking alcohol only in moderation, increasing the amount of fruits and vegetables in our diet, reducing added sugars consumption and not smoking is a pretty good start. A healthy lifestyle, combined with the control of blood pressure and lipid levels across the population, is the first step we need to take if we want to prevent probably at least half of the heart attacks and Type 2 diabetes cases out there. So in that arena, my research focuses more specifically on the impact of exercise and different dietary patterns or interventions on HDL-cholesterol levels, and also on the functionality of this lipoprotein subclass.

That being said, we should keep in mind that there are some individuals where even if they live a healthy lifestyle and do everything they are told by their doctors, personal trainers or dieticians they may already cope with, or one day develop, chronic diseases, most likely because they have a family history of CVD and have inherited CVD risk factors. This is the case, for example, for patients with familial hypercholesterolemia (and there are a lot of them in Quebec and in Canada as a whole, up to 1 in 200) and is also true for patients with high levels of an emerging risk factor for heart disease, stroke and aortic stenosis called lipoprotein(a). I have the privilege of working with the lipoprotein(a) Foundation and a lot of my work is devoted to raising awareness for this risk factor and trying to find a cure for the 15–20% of the population that have high levels of lipoprotein(a) because they have inherited it from a parent.

## Q What research are you conducting at the moment & how might it affect the clinic?

We have several ongoing studies here at the Québec Heart and Lung Institute that aim to document the impact of a healthy lifestyle on glycemic control, body fat distribution, cardiorespiratory fitness and atherosclerosis burden. Along with graduate students, we are measuring the impact of these lifestyle changes on parameters of the lipoprotein–lipid profile in patients with heart disease, including the functionality of HDL particles. We hope to provide evidence that the best way to improve HDL function is by eating a healthy diet and exercising regularly. We also have ongoing basic science studies documenting the impact of HDL particles on the pancreatic β cells and the impact of lipoprotein(a) on the calcification of valvular interstitial cells.

## Q What do you think is the biggest challenge facing your field today?

Well, I hate to state the obvious here but the challenge I face is similar to that of basically all the young investigators out there. We face important funding issues in these difficult economic times. In Canada, in the United States and Europe, success rates for receiving major funding from organizations have dropped, in many cases, under the 10% bar, which is barely enough to support senior and well-established investigators, so it does not leave much for those of us closer to the beginning of their research careers.

However, on a more global perspective, I also think that the budgets for healthcare are increasingly supporting efforts aiming at curing diseases rather than preventing them. I think our biggest challenge as scientists will be to convince governments, funding agencies and the private sector to invest in the prevention of chronic disease and help researchers develop the required knowledge from the basic mechanisms of diseases to the identification of risk factors and the genes that cause disease, and to develop the tools that will be needed to eradicate CVDs and Type 2 diabetes. In other words, we need to convince the stakeholders that investing in prevention now could save us a lot of money in the long run.

## Q If you had unlimited resources, what research would you perform & why?

I would still be in the CVD prevention business, no doubt about that. Our problem is that most of the data that we have on lifestyle-related CVD prevention come from epidemiological or observational studies. If I had unlimited resources, there is no doubt in my mind that I would use the funds to prove once and for all, in a large-scale randomized clinical trial, that people who are physically active and eat a healthy and balanced diet with unlimited access to physical activity experts and dieticians demonstrate a significantly lower incidence of CVD. I think it would help us understand precisely how many heart attacks or expensive heart-related procedures we can prevent if we do everything in our power to convince the public to live an active and healthy lifestyle.

I am a big believer in personalized and precision medicine and was very happy to hear President Obama in the last state of the union talk about how important it is to give the right treatment to the right patient and how much more research is needed in this area. I believe this is likely to be true for interventional procedures and for drugs, but it is also likely to be true for what we eat and how we exercise. I would use such a trial to identify the determinants of health from an environmental perspective, but also from a genomics and metabolomics perspective, hoping that we can one day provide personal advice on healthy living based on people's genetic makeup and environments.

## Q Where would you like to see the field in 10 years’ time?

First and foremost, I hope that within the next 10 years a global surveillance system will be set up so that we know before birth whether someone has high odds of having inherited CVD, in the hope that we will by then have better treatment for lipid disorders such as familial hypercholesterolemia and familial combined hyperlipidemia, and for patients with high lipoprotein(a).

For the rest of the population that does not have inherited risk factors for CVD, I hope that the healthcare systems will do much more to convince the population to live a healthy lifestyle. For instance, this could be by facilitating a greater access to dieticians and physical activity experts. I think this change has to come from doctors and other health professionals who should hand out prescriptions for physical activity and a healthy diet, but this change also has to come from other stakeholders such as those who design our neighborhoods, cities and the environment we ought to exercise in. It also has to come from teachers and parents who should act as role models and encourage children to perform at least 1 h of physical activity per day, for example.

In other words, I think we need do de-normalize physical inactivity and poor diets and that is the only way we shall ever be able to lower heart disease-related mortality.

## Q You seem very keen on social media. How do think social media can aid research?

Indeed I do use Twitter and Facebook and I am not afraid to say that it has significantly helped me a lot thus far in my research. It has literally changed the way I consume scientific literature. By following the accounts of scientific journals and by following your peers on social media, you can see that a paper in your field has been published the minute it is published. Social media is a great tool to connect with researchers around the world in your field without having to travel; it really brings the research community closer together. I think being active on social media is also excellent to promote your own research and get in touch with potential students, especially, in a time where the recruitment of talented graduate students has become a bit more difficult. Finally, I think it is helping us democratize our research and share our results or our thoughts directly with the public, without any filters.

## Q Finally, do you have any other thoughts for our readers?

Well, I just want to say that I think it is a great time to do research in preventive medicine, and that I am very excited to be part of the Editorial Board of *Future Science Open Access*. I am looking forward to helping it on its way to become a very dynamic, up-and-coming open access journal that will gain in popularity and I will be encouraging our readers to submit to it!

You can follow Benoit and *Future Science Open Access* on twitter at @ArsenaultBenoit and @fsgfso.

